# Feasibility of ice sheet conservation using seabed anchored curtains

**DOI:** 10.1093/pnasnexus/pgad053

**Published:** 2023-03-28

**Authors:** Bowie Keefer, Michael Wolovick, John C Moore

**Affiliations:** Adjunct, Clean Energy Research Centre, University of British Columbia, 2329 West Mall, Vancouver BC V6T 1Z4, Canada; College of Global Change and Earth Systems Science, Beijing Normal University, 19 Xinjiekouwai St, Haidian District, Beijing 100875, China; Glaciology Section, Alfred-Wegener-Institut Helmholtz-Zentrum für Polar- und Meeresforschung, Bremerhaven, Germany; College of Global Change and Earth Systems Science, Beijing Normal University, 19 Xinjiekouwai St, Haidian District, Beijing 100875, China; Arctic Center, University of Lapland, Pohjoisranta 4, 96200 Rovaniemi, Finland

## Abstract

Sea level rise is expected to be rapid and extremely damaging to coastal communities and infrastructure, with unavoidable losses and coastal protection costs in the tens of billions per year. Retreat of the Thwaites and Pine Island Glaciers is likely already in an unstable regime as their oceanic fronts are ablated by deep intruding layers of relatively warm seawater. Warm water can be blocked from reaching the grounding line by thin flexible buoyant curtains anchored to the seabed. The consequent reduction in ice shelf melting could result in increased ice sheet buttressing as the shelf makes contact with seabed highs. Flexible curtains are less costly than solid artificial barriers, more robust against iceberg collisions, and easier to repair or remove in the event of unforeseen side effects. We illustrate the technical viability of this approach by considering curtain design concepts that should withstand oceanographic forces, and feasible methods of installation. Suitable materials are commonly available. Installation of a seabed curtain in temperate ocean waters would be entirely within the capabilities of existing offshore and deep ocean construction techniques. Installing in polar waters presents severe challenges from icebergs, harsh weather, and brief working seasons, which can however, be overcome with present-day technology. An 80 km long curtain installed in 600 m deep waters on alluvial sediments could help stabilize Pine Island and Thwaites glaciers over the next few centuries at much lower cost ($40–80 billion + $1–2 billion/yr maintenance) than the global coastline protection (∼$40 billion/yr) needed due to their collapse.

**Significance statement**

Sea level rise is probably the single biggest impact of climate warming and difficult to prevent given the huge reservoir of heat in the global ocean. The largest concern for rapid sea level rise is the collapse of parts of the West Antarctic ice sheet where ocean melting has already destabilized the equivalent of a meter or more of global sea rise. We propose a system of flexible barriers to limit access of warm deep ocean currents that would allow the vulnerable ice shelves to thicken, reground, and buttress the inland ice. Despite the difficulties and risks resulting from severe ice conditions, these barriers could be installed with existing technology at lower cost, and better globally equity than coastal protection.

## Introduction

Concern that a warming climate could destabilize the primarily marine-based West Antarctic Ice Sheet (WAIS) began in the 1970s (1), and has grown with the understanding of the Marine Ice Sheet Instability (MISI), the dynamic instability created by a marine-based ice sheet whose bed deepens inland (2–4). Under MISI, the grounding line of a marine ice sheet is generally unstable on a retrograde slope (5) unless stabilized by buttressing from floating ice shelves (6) by temporal and spatial variability in basal drag (7), or by gravitational and isostatic effects (8).

Ocean thermal forcing controls both the onset and the rate of MISI collapse ((9) and references therein; (10)). Important outlet glaciers in both Greenland and Antarctica face oceanic boundary conditions characterized by warm salty waters underneath cold fresh waters. Where these deep warm waters reach the grounding line, they trigger high basal melt rates on floating ice shelves, thus reducing the buttressing force that the shelves exert on the grounded ice sheet and accelerating retreat. Warm Circumpolar Deep Water (CDW) is the proximate cause of WAIS mass loss (9). Some work suggests that the grounding line of WAIS in the Amundsen Sea sector may be in the beginning stages of a MISI collapse (11–13), but other work suggests it may not have reached that point (14). The current scientific consensus as embodied in the latest IPCC report (15; section 9.4.2.1) is that the observed retreat in the Amundsen sector is compatible with, but not unequivocally indicative of, an ongoing MISI collapse. Regardless of whether or not MISI has definitively begun, it remains a source of deep uncertainty in projections of future sea level rise, with the potential to substantially increase the upper bound of high-impact but low-probability sea level scenarios. Because MISI is a nonlinear instability problem with substantial hysteresis (5), once it begins it may lead to a collapse or partial collapse of the WAIS at rate that poses a serious threat to coastal communities worldwide, regardless of subsequent emissions reductions. The Precautionary Principle or Approach, that has been the foundation of environmental law for decades, specifically states that scientific certainty is not required before action starts, and in any case any engineering solution would require at least a full decade of study before any implementation.

Methods of blocking warm water and increasing buttressing have been proposed (16, 17), however building rigid fixed structures that could plausibly withstand iceberg impacts or ice shelf re-grounding would be technically difficult and extremely expensive. Reducing warm water access to the ice shelf base is more feasible, and may be sufficient to allow the ice to thicken and reground on natural subsea topographic seabed highs that recently buttressed the shelf (18). This paper outlines how deep warm and saline waters intruding beneath the shelf can be blocked by flexible barriers or seabed attached curtains designed to bend and deflect safely under impinging icebergs.

**Fig. 1.**
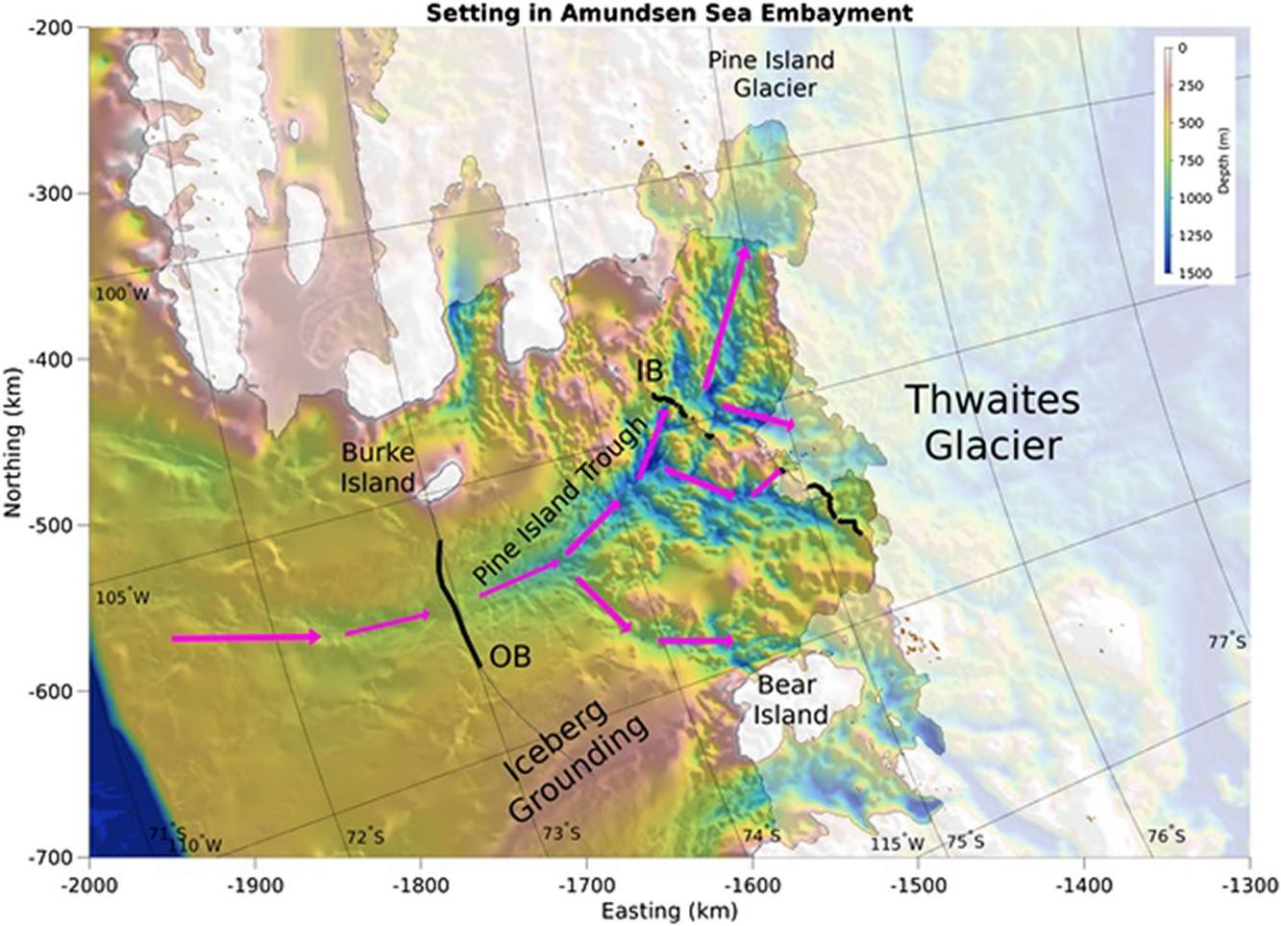
Bathymetric setting in the Amundsen Sea Embayment. Map shows hill-shaded bathymetry from BedMachine Antarctica (19) with white lines for the grounding line and ice front. Thick black lines show possible Outer Bay (OB) and Inner Bay (IB) routes under consideration. Arrows show ingress routes of warm deep waters. Typical iceberg paths cross the stranding location north of Bear Island (20).

We explore conceptual design and installation approaches at the scale of a large but achievable civil engineering project, with the potential to prevent, delay, or even gradually reverse marine ice sheet collapse of the Pine Island and Thwaites glaciers (Fig. 1). We consider the oceanographic forces that curtains attached to the seabed must withstand and hence the basic mass and extent of structures that could be used to potentially stabilize vulnerable outlet glaciers.

While this preliminary analysis supports the basic feasibility of the concept, much further detailed study will be needed to investigate fluid-structural interactions and changes in ocean circulation and sea ice resulting from curtain deployment, in parallel with engineering design and development of installation techniques.

## Seabed anchored curtain concepts

Seabed anchored curtains will be buoyantly tensioned, flexible barriers to block the progress of deep warm water towards the grounding line. They will not provide any buttressing of ice fronts. The curtains are attached to the seabed and stretched upward in the water column by their own buoyancy (Table S1; Supplementary Material: Oceanographic Load Model). To minimize potential damage, they would comprise overlapping flexible panels, which would bend and slide under impinging icebergs. The principle of underwater curtains has been demonstrated at relevant scale by large “temperature control curtains” (21–23) of rubberized fabric used in thermally stratified hydroelectric reservoirs for moderating water temperatures in downstream discharge flows.

**Fig. 2.**
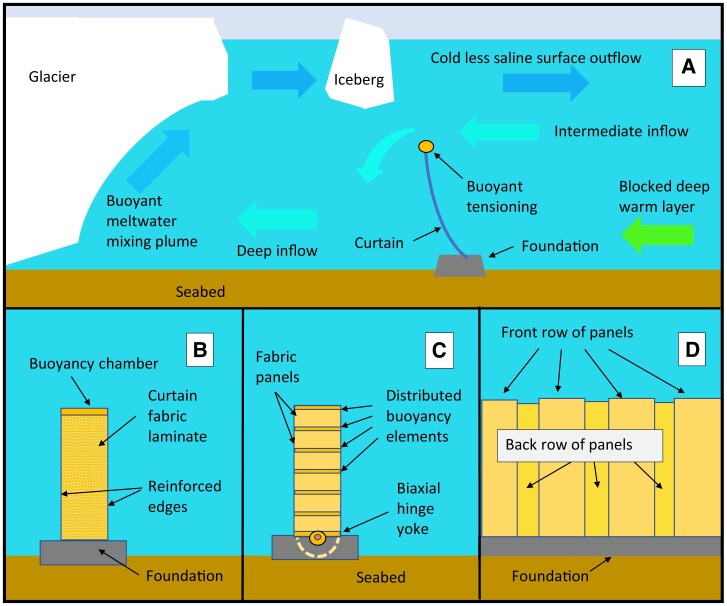
Seabed curtain schematic (not to scale). A) Exchange flow over the curtain. Front view of a single curtain panel with buoyancy concentrated at the top, B), or distributed along the height (C) showing how the curtain panels could be pivoted to allow transverse sway. Sets of panels (D) joined to make a long curtain with overlapping front and back rows to accommodate locally varying forces.

The curtains include two main components: their foundation, and the curtain itself (Fig. 2). The foundation must withstand the horizontal load created by hydrodynamic and hydrostatic forces on the curtain, as well as the net vertical uplift load produced by the curtain buoyancy. The foundation may be effectively permanent once installed, while the curtains would be detachable for repair or replacement in the event of damage, or even removal if the intervention runs into unforeseen consequences and needs to be dismantled.

The seabed curtain panels are designed to tilt and bend in response to changing ocean loads. As tides and currents change, the curtain will passively adjust its lean angle and curvature so that the horizontal forces dictated by oceanographic conditions are balanced by the vertical buoyancy force produced by the curtain itself. The curtains are flexible and will passively lean closer to the horizontal under heavier loads from stronger ocean currents or steeper density stratification, avoiding catastrophic failure. Higher loads would cause a gradual reduction in effectiveness as increasing volumes of warm water are mixed over the curtain, which can rebound when those loads are reduced. There must be enough buoyancy in the curtain to keep it sufficiently upright to effectively block enough warm water under realistic ranges of oceanographic loading. The vertical buoyancy force must thus support the typical oceanographic forces on the curtain at its target height above the seabed. Larger oceanographic forces would require increased curtain buoyancy to achieve a desired height, while increased buoyancy in turn requires greater curtain tensile strength and stronger foundations. The curtain panels must bend frontally under changing oceanographic loading and potentially, iceberg collisions, either by flexure or by mechanical hinges between the panels and the foundations (Fig. 2C).

## Oceanographic loads

The oceanographic forces on the curtain include differential hydrostatic pressure resulting from contrasting vertical density gradients on opposite sides of the curtain, and hydrodynamic drag resulting from fluid flow across or along the curtain. As the curtain will be resisting overflow from the deeper and higher density CDW ocean layer, the hydrostatic and hydrodynamic loads will both typically be directed towards the ice front across the curtain. Once the seabed anchored curtain has been installed, the vertical density contrasts and resulting hydrostatic loads are expected to increase due to freshening on the sheltered glacier side of the curtain. At the same time, hydrodynamic loads from flow across the curtain will be changed as reductions in the melt rate reduce the driving force for the overturning circulation in front of the glacier, and volumetric exchange flows would be reduced by curtain blocking action while local currents may be increased in the constricted aperture above the curtain. Thicker winter sea ice may also be expected because of reduced heat release from CDW import.

The stratification between the cold meltwater-freshened surface layer and the deeper layer of relatively higher temperature and salinity leads to a pycnocline, typically 200–300 m thick (Fig. S1; Supplementary Material: Oceanographic Load Model) and closely coinciding with the thermocline, that varies under wind, tidal, and external current forcings (24). There will be strong shear and eddies associated with the exchange flow between the outgoing cold surface layer and the incoming more saline deep layer.

The dominant flow pattern over the curtain will be the exchange flow between deeper seawater overflowing the curtain toward the glacier front, and an outward surface flow of lower density seawater freshened by meltwater and floating ice. This exchange flow is driven from the glacier side by the buoyant plume of subglacial drainage turbulently entraining seawater and meltwater from the glacier front, and from the continental shelf margin by variations in upwelling related to changing wind forcing and Ekman pumping. The exchange flow is resisted by form drag across seabed obstacles, seabed skin friction, shear friction between the exchange flow layers, wave drag from internal wave generation, and dissipation from turbulent mixing.

The steady drag force across the curtain will largely result from flow separation on its lee side, with a minor contribution from skin friction on the curtain front. The drag force will be approximately quadratic with current velocity and linear with curtain height. This drag force is self-limiting for the following reasons: (1) while the drag varies quadratically with flow velocity, the volumetric flow itself is opposed and thus reduced by greater drag, (2) successful blocking of deeper warm water intrusion will reduce the volume of meltwater buoyantly forcing the exchange flow circulation, and (3) a curtain will reduce the density contrast driving the buoyant meltwater plume.

While presently observed ocean currents in deep troughs near Thwaites and Pine Island Glaciers are of the order of 10 cm s^−1^ and mostly less than 20 cm s^−1^ (25), much larger local current velocities may result from emplacement of a seabed anchored curtain and particularly during its installation. The maximum inflow velocity will be constrained by hydraulic criticality conditions (26) for stratified exchange flows. Installing the curtain will reduce both inward and outward volumetric flows, while increasing flow velocity over the curtain crest corresponding to the reduction of cross-sectional flow area for the inward flow.

The frontal drag resulting from the maximum inflow velocity will be less than the hydrostatic load due to density differences if the curtain height is sufficiently tall relative to water depth. Relatively low drag would arise for subcritical flow expected over most of the curtain length when the artificial barrier is longer than the Rossby radius. Considerably larger drag forces may apply locally with critical flow at one end of the curtain, or unchoked bypass flow around the ends of the curtain or the edges of any breach in the curtain. The expected dependence of net force on curtain height would be roughly linear for the hydrodynamic drag component, but much steeper for the hydrostatic component arising from density differences across the curtain. Table S1 gives forces from our loading model on curtains of different height with oceanographic properties based on observations in the Amundsen Sea. In this example, total horizontal force is 5.8 kN/m for a 100 m high curtain, rising to 370 kN/m for a curtain 500 m high. It is evident that taller curtains are much more challenging than shorter ones (Fig. S2).

## Dynamical effects

The artificial sills provided by large-scale curtain installations in the Amundsen Sea will change circulation patterns and pycnocline profiles. By design, less warm CDW would flow under the ice shelves, which would thicken until a new steady-state equilibrium is established. A wide curtain barrier would consequently reduce the outflowing fluxes of meltwater and floating ice, flattening the hydraulic gradient of the pycnocline from the continental shelf edge to the curtain location on the seaward side of the glacier fronts. It is possible that a reduction in the overturning circulation caused by curtain installation might also cause a reduction in the observed east-west gradient (24) in the pycnocline across Pine Island Trough (Fig. S3). Time-variable components of the ocean circulation, predominantly tides and eddies, would induce transient loads on the curtain, while these deeply submerged curtains are well isolated from ocean surface wave effects.

Natural circulation patterns in the Amundsen Sea are highly variable in both time and space. A central gyre is sometimes prominent in Pine Island Bay (24, 27), and seasonal polynyas may appear along the east coast extending northward from Pine Island Glacier (28). Numerical ocean modeling (29–31) and field observations in Pine Island Bay (31, 32), suggest that large natural sill barriers across such wide bays can establish full-width eddies on opposite sides of the barrier, with tangential flow along the barrier corresponding to cyclonic flow on the landward side and upsetting of geostrophic flow on the ocean side after the sill has blocked inward barotropic flow.

In the southern hemisphere, the expected tangential flow along the curtain would be directed from west to east (Fig. S3), possibly at somewhat higher velocities than the frontally directed flow across the curtain and may cause some aspiration of deeper and warmer water, which might be countered locally by raising the curtain height. With tangential flow along the curtain, aspiration by Ekman advection will be arrested by the balance between buoyancy and Coriolis forces, with the frontal steepness of the curtains being highly advantageous compared with gently sloping natural sills.

There is ample evidence that natural sills do block deeper layers to a large extent, as least in straight channels whose width does not greatly exceed the Rossby radius of deformation. There is also evidence of partial aspiration of deeper water layers from below the sill crest as observed in the Strait of Gibraltar (33) and elsewhere (34). The vertical extent of aspiration will be determined by the balance between kinetic energy of the frontal flow and buoyancy potential energy, thus by the quotient of current velocity divided by the buoyancy frequency.

While the basic curtain objective is simply to block deep water ingress from beneath a target depth, the concept can also be extended to “guide vanes” or “training walls” to discipline current patterns and eddies in support of the main curtain blocking function. We potentially have a rather versatile toolbox for exploring possible performance enhancements (albeit typically with large cost penalties) by such measures as (1) simply raising curtain height with more buoyant tensioning, (2) installing two parallel curtains approximately a Rossby radius (∼5 km) apart, or (3) installing short “training wall” curtain segments orthogonal to the main curtain at suitable intervals to suppress eddy-generated tangential flows.

Fluid-structural interactions will include multiple oscillatory and traveling wave modes, and possible resonant or chaotic instabilities. Excessive oscillations could cause progressive degradation by flexural fatigue, chafing damage between panels, and bearing wear. Such oscillations and consequent degradation modes may be mitigated by several possible strategies such as (1) adjusting panel tension by changing net buoyancy; (2) optimizing the vertical distribution of curtain buoyancy and the resulting curtain curvature; (3) adjusting panel flexural stiffness and/or damping; (4) rounding panel tops to delay hydrodynamic separation; (5) shaping panel tops with spoilers to disrupt vortex shedding; (6) adding slots near the top of the curtain for boundary layer control; (7) adding textured drag elements to the curtain to increase damping and added mass; and (8) configuring buoyancy elements at the top of the curtain to act as dynamic vibration absorbers (see Supplementary Material: Curtain Design Considerations).

## Seabed anchored curtain design

Curtain panels will have the following functional components (Fig. 2): buoyancy elements, vertical tensile members, a structural lattice, and low permeability fabric covering gaps in the lattice. Curtain buoyancy may be concentrated at the top or distributed either at lumped intervals or uniformly along the vertical length of the panels. The buoyancy elements must withstand collapse or bursting under changes of hydrostatic pressure between the surface and seabed depth; and to be stable, over a design life of at least 25 years, against saltwater corrosion and buoyancy loss by fluid leakage or diffusion. Polymeric composites based on glass or carbon fibers can meet all requirements with advantages of near neutral buoyancy of the structural material, and a well-proven subsea record. Alternatives include filament-wound composite cylindrical pipes with closed ends, blocks of syntactic foam or corrosion-resistant composite pressure vessels.

Tensile members need to support the oceanographic horizontal loads at a suitable lean angle of the curtain (Fig. 2). These could run vertically up the edges of the panels and at evenly spaced intermediate positions. A laminated high modulus polyethylene (HMPE) panel structure may be designed to support all or part of its own tensile loads. Alternatively, vertically oriented composite pipes could combine distributed buoyancy and axial tensile strength for fabric curtains with considerable bending flexibility and abrasion resistance. Commercial fiberglass epoxy pipe of 0.15 m outside diameter used for subsea flowlines can support an ultimate tensile load of 260 kN (35), allowing curtain installations up to about 300 m height without further reinforcement (Table S1). Taller curtains would require additional tensile strap reinforcement in their deeper portions. Stiffening braces may be provided as horizontally oriented battens at intervals along the panel length, using composite pipes which also contribute to needed panel buoyancy. Panels may be hinged at the battens along their length to enable deployment from a folded stowage position, or alternatively may be delivered as coiled rolls.

**Fig. 3.**
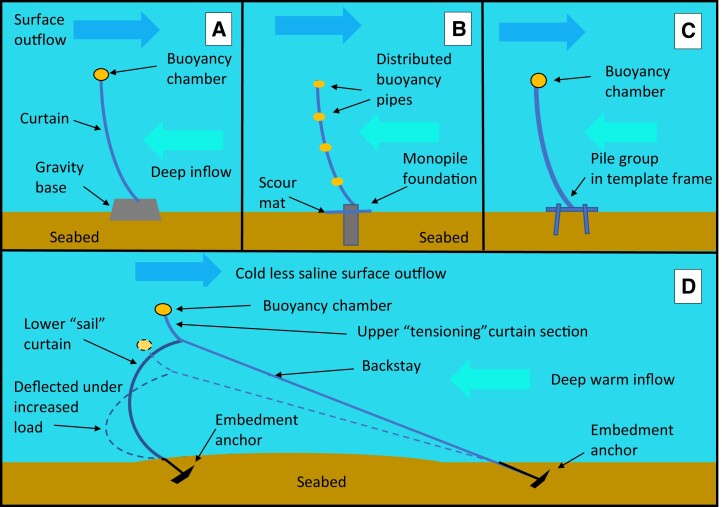
Foundation configurations for alluvial seabeds. A) simple gravity modules, B) individual monopiles under the hinge pivot of each panel and with scour protection in the gaps between monopiles, C) groups of piles driven through template frame modules, D) compound curtains with a buoyant upper section tensioning a lower “sail” curtain section with further tensioning from backstays. Buoyancy elements may be arranged as chambers at the curtain top (A, C, D) or as horizontal glass epoxy pipe stiffeners at intervals along the panels (B).

Diverse seabed foundations and anchoring techniques (36) are used for offshore platforms and wind turbines dependent on geotechnical seabed conditions and orientation of tensile loads (Fig. 3). While curtain hydrostatic and hydrodynamic loads are predominantly horizontal, vertical uplift from necessary buoyancy may be more difficult to handle. Gravity foundations (Fig. 3A) must have a submerged weight exceeding the maximum uplift force by a sufficient margin to prevent sliding under the horizontal forces. The rapidly climbing uplift tension forces with curtain height (Table S1) may make gravity foundations for any extremely tall curtain sections impracticably large and heavy for ocean transportation.

Monopiles foundations (Fig. 3B) have been successfully used for offshore wind turbines, and have the structural elegance that the panel pivots can be attached directly to the pile head. Bypass leakage and scour between the piles can be blocked by appropriate mats. Grouped piles (Fig. 3C) with template frames have been used to withstand the large uplift forces of deep-water tension leg platforms. Compound curtain panel designs with a relatively light upper section and a stronger lower section manage the much larger differential pressure as in Fig. 3D offer reduced vertical uplift forces on the foundations and a smaller need for buoyancy elements in the panels and may be suitable for clay or sand soils. The buoyantly tensioned upper panel sections provide necessary tensioning for proper deployment of the lower sections. There is some risk of snagging by icebergs from the ocean side over the backstays and risk of damage must be weighed against ease of replacement, seabed conditions, and hydrodynamic drag loads.

## Fabrication and installation

Punta Arenas is the closest location to the Amundsen Sea for a fabrication, assembly or staging plant. Towing large floating modules across 2,500 km of ocean south of Cape Horn, and then bringing them through sea ice in the Amundsen Sea before lowering them to the seabed with great precision, is non-trivial with considerable operational risk. Fortunately, the most advanced polar icebreakers are now typically double-ended ships (37) designed for excellent sea-keeping characteristics in the forward direction and powerful icebreaking capability in the reverse direction. These icebreakers have steerable electrically powered azimuthal propulsion units (38) which facilitate precise dynamic positioning for subsea operations and may be used to clear ice around the ship's working area.

Semi-submersible heavy lift ships and transporter vessels are capable of carrying very large loads such as entire ships or offshore petroleum platforms. The load is floated on and off the flooded cargo deck by ballasting the ship. All ships would need to be ice-strengthened to Lloyds class 1A. Amundsen Sea ice conditions vary seasonally, and while lacking the thick pressure ridges of overlapped floes seen in the Arctic Ocean, have large icebergs that must be worked around (Supplementary material: Installation Methods).

Large diameter piles have been driven in granular or glacial moraine soils at much greater ocean depths (36) than any part of the Amundsen Sea using vibratory or impact subsea piledriving equipment with self-contained hydraulic power packs energized by umbilical power connections from the surface. Small diameter piles can be driven by undersea impact hammers or vibratory drivers or may be torqued into place as screw piles after acoustic mapping of obstructions such as buried boulders in glacial till. Piles may be drilled and grouted into bedrock. Embedment anchors may be installed rapidly by the largely horizontal pulling force exerted by powerful anchor handling vessels, or may be gravity driven at terminal velocity by dropping from ∼200 m above the bed.

## Challenges and risks

### Icebergs

During installation, icebergs may block access to some of the curtain route. The risk may be mitigated somewhat by having multiple working locations along the curtain route that can be selected based on evolving ice conditions; nonetheless, some fraction of the potential working time will be lost to iceberg disruptions. Once the curtain is installed, iceberg risks can be separated into those that collide with the curtain panels without impacting the foundations, and very deep draft icebergs that may disrupt the curtain foundations and perhaps plough the seabed.

Wolovick et al. (39; companion paper) argue that a curtain top depth greater than 500 m would provide optimal cost-benefit. The draft of Thwaites ice shelf and Pine Island Glaciers are about 300–500 m (19) meaning that most icebergs will pass overhead without contacting the curtain. By design concept, the curtain panels accommodate iceberg collisions by bending and flexing to slide under the berg. Deformation of the curtain panels around the berg will create gaps that allow some warm water to temporarily slip through the curtain.

The curtain material should be smooth, slippery, and highly resistant to abrasion or cuts. These material properties should enhance the ability of the panels to avoid serious damage from iceberg encounters not impacting the curtain foundations. As the curtain will already be somewhat porous because of overlapping panels (Fig. 2), its functionality should withstand minor tears.

The deepest portions of contemplated curtain routes are relatively safe from foundation ploughing by the deepest draft icebergs presently being discharged by Thwaites and Pine Island glaciers. However, the shallower flanks of curtain routes will be exposed to iceberg grounding and ploughing events. Defensive measures might take the form of installing the curtain foundations in a wide trench, with the spoil used as fill for sufficiently reinforced and armored berms on each side of the trench.

Icebergs from Pine Island and Thwaites Glaciers tend to move northwards in a relatively narrow track along the west side of Pine Island trough (20) and frequently ground on the central Amundsen Sea ridge extending northwards from Bear Island (Fig. 1). Capsizing and breakup of stranded icebergs on that ridge may present a threat to the western end of a mid-shelf seabed curtain across the trough. A large mid-shelf curtain installation may impact current patterns and iceberg movement; persistent northward barotropic flow past Bear Ridge may allow the western end of that curtain to be shortened with cost savings and reduced risk of iceberg impacts. Smaller icebergs from the Abbott ice shelf and the Bellingshausen Sea may drift southwards into the area around Burke Island before being swept away northwards along the west flank of the trough (20). A mid-shelf curtain may reduce southward barotropic flow approaching Burke Island, thus reducing iceberg encounter frequency in that vicinity.

### Changing loads

Curtain-induced reductions in exchange flow current velocities are likely to increase the density difference across the curtain by freshening the interior waters (Fig. S3). Peaks in dynamic loads will arise from tsunamis, seiches, and internal waves caused by iceberg calving and rollover. If greater buoyancy and tension were required to achieve desired blocking effectiveness, capital costs would also increase. Local curtain height variations or installing extra curtain sections to function as current guide vanes could mitigate peak loads.

### Degradation and maintenance

Flexural fatigue and chafing wear will accumulate over time from movement and oscillations of the curtains. Curtain maintenance plans should include pre-positioning of spare curtain components and operational equipment for inspection, servicing, and emergency installation. Individual curtain panels can be removed from the foundations for easy replacement, but replacement of foundation modules would be more difficult and expensive. Based on the material properties and history of use of the curtain materials considered above, a design life target of 25 years for the curtain panels and 100 years for the foundation modules can be expected. However, detailed fluid-structural dynamic simulations and tank tests of curtain designs immersed in unsteady exchange flows are needed to optimize resilience against oscillations and deflections that ultimately will determine the achievable life.

### Accelerated ice shelf breakup

An increased flux of icebergs, including deeper draft icebergs due to rapid loss of ice shelves and glaciers in the Amundsen Sea embayment would make curtain installation more difficult. The speed and consequences of rapid disintegration are essentially unknown at present, but retreating ice fronts would likely slow re-grounding.

### Marine ecology

Curtain construction and changes to ocean circulation patterns will impact regional marine ecology, but these will also be impacted by dramatic retreat of the ice sheet. Although the objective is to maintain the ocean and glacier system at the status quo, the possible deployment will be governed by the Madrid Protocol (40; https://www.ats.aq/e/ep.htm).

## Technical and economic practicability

At this stage, engineering design concepts for subsea anchored curtains are insufficiently developed and too little is known about seabed conditions for total costs of fabrication and installation to be estimated with great confidence. However, hypothetical installation of structures similar to our seabed curtains in temperate ocean waters is within the capabilities of present offshore and deep ocean construction techniques. Pipelines are laid and heavy platform foundations are installed in much deeper water than here contemplated, while massive structures are routinely moved to or from the seabed. Complex tasks are performed by remote operated submersible vehicles. While surface and subsea research and reconnaissance missions are increasingly performed in arctic waters, the extension of full ocean engineering capabilities to the margins of the polar ice sheets will be very challenging. Specialized equipment including icebreakers with heavy lift and submersible deployment capabilities will be needed. Polar night, adverse weather and iceberg movements will disrupt work.

Simultaneous stabilization of both Thwaites and Pine Island Glaciers may be achieved by a long subsea curtain installation taking advantage of existing partial sills in Pine Island Bay, located either on a dissected ridge across the inner bay near the present ice shelf front, or in a mid-shelf location of Pine Island Trough at approximately 73^°^ S latitude (Fig. 1)—and see Wolovick et al. (39; companion paper) for detailed analysis of curtain siting options. While the highest-leverage component of the inner bay route is less than 5 km long, for this analysis we conservatively assume an overall length of 80 km and a water depth exceeding 600 m, roughly consistent with both the mid-shelf route and the total inner bay route (Fig. 1).

The seabed at the mid-shelf location is glacially sourced sedimentary deposits making for easier engineering design and installation than on the glacially carved bedrock of the inner bay route. Allowing for the costs of curtain and foundation materials, fabrication costs, the engineering and manufacturing costs of necessary specialized equipment, construction of icebreakers and robustly ice-reinforced heavy lift ships, seabed geotechnical investigations, seabed preparation using remotely operated subsea machinery, and installation of foundations and curtains during short and frequently interrupted working seasons, the decade of construction for a mid-shelf subsea curtain protecting both Pine Island and Thwaites Glaciers may cost $4–8 b/yr with ongoing maintenance in the range of US $1–2 b/yr (Supplementary material: Costs). This represents a cost of about $5–10 per person per year for the 200 million people who are projected to move away from flooded coasts (41). These costs can be compared with projected sea dike capital investment at about $40 billion/year for a sea level rise of 1 m (42). These coastal defenses would also entail the environmental impact of tens of thousands of km of infrastructure construction.

## Conclusions

There is a real risk of multi-meter sea level rises over the next few hundred years. The feasibility of coastal protection depends on individual country wealth and tends to be more expensive as a fraction of GDP for relatively poorer countries, meaning that inequalities are likely to be exacerbated by sea level rise (42). The Developing World will undoubtedly face the greatest difficulties, including existential threats to small island states, despite having generated tiny greenhouse gas emissions. Preservation of substantially intact polar ice sheets seems to be the most equitable, as well as the most cost-effective defense against rising seas.

We recommend the seabed anchored curtain concept for high priority feasibility evaluation and development (Supplementary material: Research Priorities). The outstanding research questions to be prioritized include modeling of coupled oceanographic and ice dynamic response to deployment of curtains and analysis of fluid-structural interactions, oscillation modes, and potential instabilities. While special urgency may be attached to consideration of an extremely large curtain installation in Pine Island Bay in view of the perceived instability of Thwaites Glacier with potentially severe consequences of its collapse, an overall program to mitigate global sea level rise by stabilizing marine-terminating glaciers would certainly contemplate multiple prospective sites in Greenland and Antarctica. An orderly progression from small-scale pilot demonstration projects to larger Greenland fjords, and then to much larger Antarctica sites, would provide experience along the engineering learning curve, backed by field testing results on materials selection and manufacturing methods. The overall potential benefits of this technology can be determined by making a global inventory of potential sites and assessing the cost-effectiveness of achievable sea level rise mitigation contributions from each site.

Unlike rigid underwater barriers, curtain flexibility allows them to deform around impinging icebergs, and their modularity facilitates installation and maintenance. They can be installed using ballast controlled from the surface, and then unfolded once they are attached to the seabed. They can be removed if the intervention produces unanticipated side effects. Preliminary consideration of hydrostatic and hydrodynamic oceanographic loads indicates that simple curtain designs with available materials will be capable of providing the buoyancy and tensile strength necessary to withstand those loads. It will take considerable effort by the scientific and engineering communities to refine and demonstrate an implementable design. Without deep greenhouse gas emission cuts, it is probable that warming atmospheric temperatures would eventually destroy their effectiveness. The hundreds of years commitment to maintenance and long-term governance of sea level rise management means that governance, funding and social licence must go far beyond the 29 consultative parties to the Antarctic Treaty System (43).

## Supplementary Material

pgad053_Supplementary_Data

## Data Availability

Oceanographic datasets used are from World Ocean Database https://www.ncei.noaa.gov/products/world-ocean-database, and tables and figures are generated using the methods detailed in the Supplementary Material with this manuscript.
