# Supplementary material for: Feasibility of ice sheet conservation using seabed anchored curtains
Source: PNAS Nexus. 2023 Mar 28;2(3):pgad053. doi: 10.1093/pnasnexus/pgad053 (PMC10062297; doi:10.1093/pnasnexus/pgad053)
Supplement: pgad053_Supplementary_Data [file pgad053_supplementary_data.docx]

**Supplementary Information**

1. **Oceanographic Load Model**

Typical thermocline profiles observed in Pine Island Bay are shown in Figure S1a, and the very similar pycnocline profiles are shown in Figure S1b. For illustrative simplified load calculations, we consider a curtain cresting at the top of the pycnocline with an assumed piecewise linear profile (Figure S1c). While a hyperbolic tangent function would provide a better approximation to real dispersive flows, the following piecewise linear description is convenient for simplified preliminary calculations. The curtain has height *H* above the seabed, and a forward lean angle $\theta$ from vertical at its base. Density difference relative to the upper layers is $\Delta\rho$_o_ at the base of the curtain. The thickness of the pycnocline in deep water is Δ*z* ≡ *(H – h)*. The hydrostatic density difference will be zero at the top of the curtain, while the total pressure difference across the curtain will also include drag contributions from hydrodynamic separation of inward flow and skin friction. The assumption of a constant density difference below intermediate height *h* corresponds to the vertical gradient of density stratification on the outer side of the curtain being balanced by a similar gradient on the inner side.


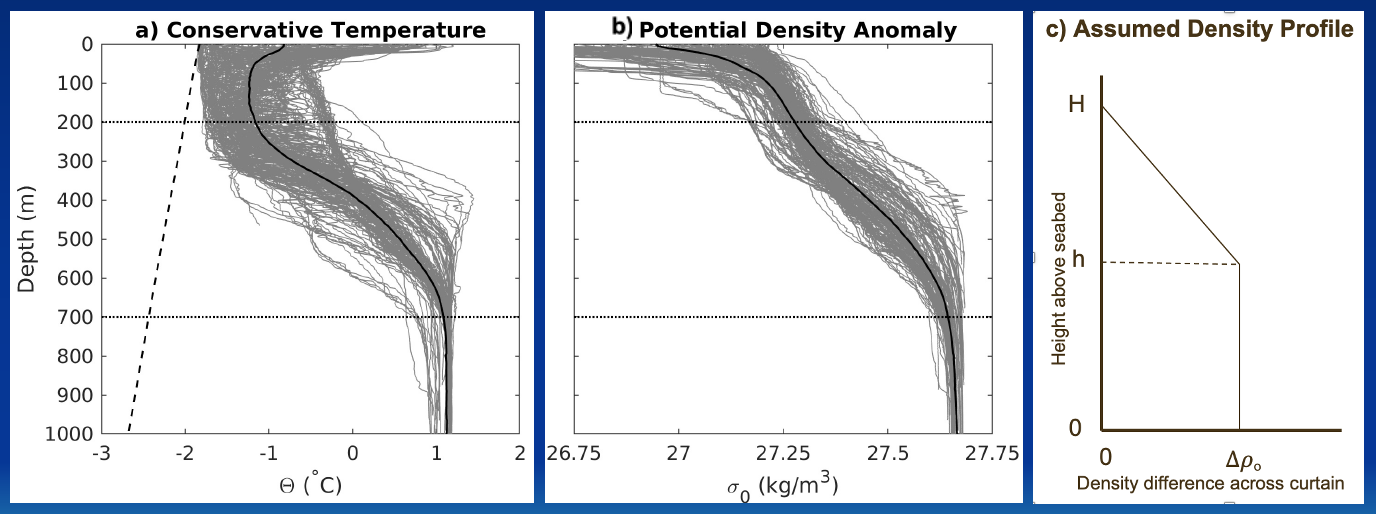


Figure S1. All CTD casts in the World Ocean Database in Amundsen Sea Embayment showing thermocline profiles of vertical temperature (a) and density (b) profiles, and (c) an assumed piece-wise linear profile of differential density.

We describe a curtain by its height vertically above the seabed and its horizontal width across a bay. Each curtain panel is characterized by its horizontal width and its length along the generators of its vertical profile, with the panel length always greater than the curtain height in the presence of finite loading from density differences or currents. Our chosen coordinates are *x* southward across the curtain, *y* eastward along the width or axis of the curtain, and *z* vertically downward from the top of the curtain. Current velocities are *u* in the *x* direction and *v* in the *y* direction.

In deep water where the thermocline is entirely above the seabed (*h >* 0), the density difference across the curtain is given by

In deep water where the thermocline is entirely above the seabed (*h >* 0), the density difference across the curtain is given by

$\Delta\rho$ *=* $\Delta\rho$*_o_ z/*Δ$z$ for *0 < z <*Δ$z$

$\Delta\rho$ *=* $\Delta\rho$*_o_* for $\Delta z$ *< z < H*.

The horizontal hydrostatic force applied to the curtain per unit width is then

*F_density_ =* $\Delta\rho_{o}g\frac{(H2+Hh+h^{2})}{6}$ for deep water with *H >* Δ*z*.

In shallower water on the flanks of a trough where *H <* Δ*z*, the pycnocline will be intercepted by the seabed. Assuming the same vertical density gradient of the pycnocline as in deep water, the horizontal hydrostatic force per unit curtain width is then

*F_density_ =* $\frac{\Delta\rho_{o}gH^{3}}{6\Delta z}$

The hydrodynamic separation drag on the curtain is proportional to the momentum flux carried by the ocean current, thus to the square of current velocity over the curtain. The drag may be resolved into a frontal component bending the curtain panels fore-and-aft across the curtain axis, and a lateral component tilting panels sideways in their own plane. The frontal drag component will be dominated by form drag (including separation and wave drag components) with a minor contribution from skin friction, while the lateral drag will result mainly from skin friction. The typical frontal form drag coefficient for a solid vertical wall immersed in the boundary layer of a steady unidirectional frontally directed flow *u* would be of order $C_{D}$ ~ 1.2 (Dong et al, 2008), which would apply to barotropic flow conditions. However, a seabed curtain is typically in the stratified and highly sheared transition below surface water layer flowing in another direction. The skin friction drag coefficient for tangentially directed flow *v* may be of order $C_{F}$ ~ 0.003 (Pratt, 1986). The horizontally projected frontal drag force *F_drag_* per unit width and lateral skin friction traction $\tau$ are thus

*F_drag_ =* $\frac{C_{D}}{2}{\rho u}^{2}H$ and $\tau$ *=* $\frac{C_{F}}{2}{\rho v}^{2}$*.*

The total horizontal and frontal force per unit width is then *F_total_ = F_density_ + F_drag_.*

An example is provided in Table S1 for horizonal forces and tension (kN/m) per unit width at the base of curtains extending to crest heights *H* above the sea bed, with across panel current velocity *u =* 0.2 m/s, assumed drag coefficient$C_{D}$ = 1.2 and curtain lean angle $\theta$ = 30^o^ for the same pycnocline profile Δ*ρ* = 0.5 kg/m^3^ across a 250 m thermocline layer thickness. The curtain crest is assumed to coincide with the top of the thermocline (Fig. S1c), and the seabed is above the base of thermocline for H ≤ 250 m, as expected for flanks of curtain routes.

Table S1. Horizonal forces and tension (kN/m) at the curtain base

| Curtain height *H* (m) | 50 | 100 | 150 | 200 | 250 | 300 | 350 | 400 | 450 | 500 |
| --- | --- | --- | --- | --- | --- | --- | --- | --- | --- | --- |
| Thermocline base *h* (m) | 0 | 0 | 0 | 0 | 0 | 50 | 100 | 150 | 200 | 250 |
| Hydrostatic force, F_density_ | 0.4 | 3.3 | 11 | 26 | 51 | 88 | 137 | 198 | 272 | 358 |
| Drag force, F_drag_ | 1.2 | 2.5 | 4 | 5 | 6 | 7 | 9 | 10 | 11 | 12 |
| Horizontal force, F_total_ | 1.6 | 5.8 | 15 | 31 | 57 | 95 | 146 | 208 | 283 | 370 |
| Tension (30^o^ lean angle) | 3.3 | 11.5 | 29 | 62 | 114 | 190 | 291 | 415 | 565 | 740 |

While the above indicated drag force may be typical for most of the curtain width across the channel, the local horizontal drag force may be increased by an order of magnitude with critical hydraulic flow control that may be experienced at the western end of the curtain. Curtain tensile loads would still be dominated by the hydrostatic term for curtain heights greater than about 250 m.

The vertical length S of the curtain panels will be greater than *H*, as the resultant of base inclination angle $\theta$ and equilibrium curtain panel curvature responding to the distribution of buoyancy (Fig. S2). Normal fluctuations of the thermocline profile will of course result in changing curtain curvature. The curtain curvature may be kept fairly low if the buoyancy is distributed along the panel length, which will also reduce the tension toward the top of the panels and thus allow lighter construction of the upper panel sections. Distributed buoyancy will also reduce any tendency for curtain oscillations resulting from panel curvature.

The following analysis applies to the case that the buoyancy is concentrated at the top of an otherwise neutrally buoyant curtain (Fig. S2). It may also be applied to neutrally buoyant curtain sections between vertically separated lumped buoyancy elements. In the approximations that the curtain panels have negligible bending stiffness and that skin friction can be neglected, the radius of curvature of the panels will be the quotient of the tension *T* per unit width over the local pressure difference *ΔP* across the curtain. Defining *θ* as the inclination angle of the curtain from vertical in the *x-z* plane and S as the length coordinate of the panel, the curtain profile geometry is given by

$\frac{dS}{d\theta}$ = $\frac{T}{\Delta P}$ , $\frac{dx}{dy}$ = *tan*$\theta$ and $\frac{dS}{dy}$ = *sec* $\theta.$

The vertical dependence of *θ* as a function of *y* is readily found from the balance of forces :

$Tsin\theta$ *=* $\frac{C_{D}}{2}{\rho u}^{2}z$ + $\frac{1}{3}\frac{{\Delta\rho}_{o}}{\Delta z}gz$^2^ for *0 < z <*Δ$z$

$Tsin\theta$ *=* $\frac{C_{D}}{2}{\rho u}^{2}z$ + $\frac{1}{3}\frac{{\Delta\rho}_{o}}{\Delta z}g\Delta z$^2^ + $\frac{1}{2}\Delta\rho$*_o_* g ${(z-\Delta z)}^{2}$ for Δ$z$ *< z < H*.

If the buoyancy is concentrated at the top of the curtains, the tension in neutrally buoyant panels with very low bending stiffness will be almost constant apart from minor corrections for skin friction drag. This almost constant tension would be equal to the buoyancy applied to the top of the panels (with a possible correction for any extra drag elements also located at the top of the curtains). The curtain panels will then be highly curved, with curvature concentrated toward the bottom (Fig. S2). A limiting case is for the tension to be vertical at the top of the certain and almost horizontal at the bottom with the panel base nearly lying on the seabed. This makes the tension and imposed buoyancy both equal to the total horizontal force, and thus lower by a factor of two compared to a curtain design with distributed buoyancy and a base inclination angle $\theta$ of 30^o^.

Fig. S2 compares curtain foundation loads and the curtain deflected profile for two alternative approaches of concentrating the buoyancy at the top of the curtain or evenly distributing the buoyancy along the panel length. Concentrating the buoyancy at the top would marginally reduce the vertical uplift load component and the amount of buoyancy required for a given ratio of panel length to curtain height. Disadvantages of concentrating the buoyancy at the top of the curtain include the need for the entire panel length to handle the full tension rather than tapering down to the top, and the vulnerability of buoyancy elements at the curtain crest to damage from icebergs. Conversely, concentrated buoyancy at the curtain top may avoid long term reliability issues of small buoyancy elements and accelerated curtain chafing wear caused by protrusions along the length of the overlapping panels. Oscillation modes associated with curtain curvature may be the greatest issue to be investigated for this approach.

 
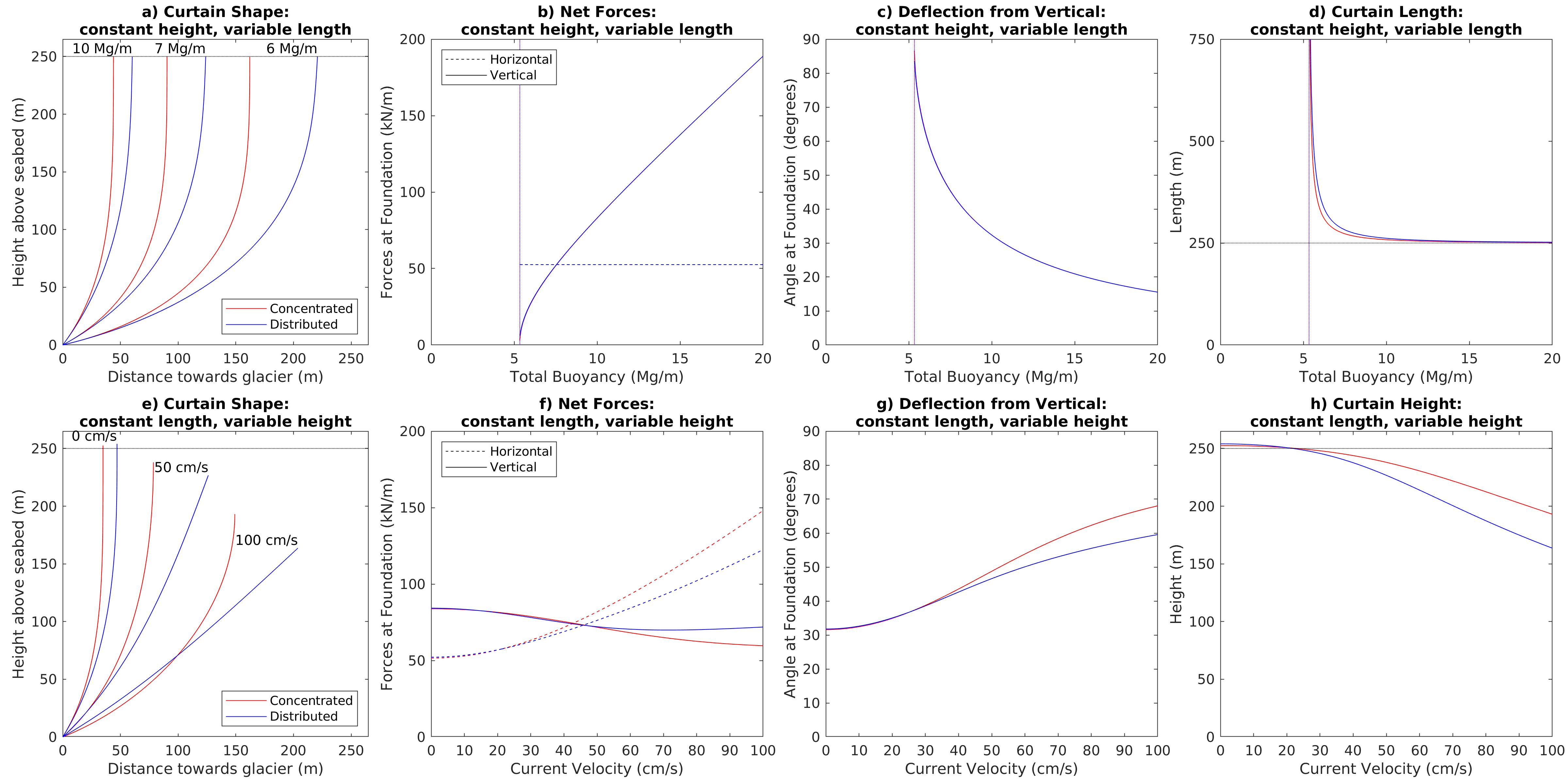
 Figure S2. Curtain geometries and loads. The top row (a-d) shows how curtain geometry changes when the top height is set at 250 m and the buoyancy and length of the curtains varies, while the bottom row (e-h) shows how curtain lean changes for fixed buoyancy and length under variable oceanographic conditions.  Thus, the top row represents the choices that might be made during curtain design (i.e., the target blocking depth and the mean oceanographic conditions are known, but we have the freedom to vary curtain length and buoyancy in order to fit that blocking depth), while the bottom row represents the variability that might be expected in curtain performance for a given curtain design under varying oceanographic loads  (i.e., length and buoyancy are now fixed, and the curtain adjusts its shape passively).  In both rows, red colors represent curtains with the buoyancy entirely concentrated at the top, while blue colors represent curtains with buoyancy evenly distributed along their length.  The first column (a,e) shows representative curtain shapes, the second column (b,f) shows tensile loads on the foundation, the third column (c,g) represents the lean angle of the curtain at the foundation, and the final column represents either the length of the curved curtain (d) or the height above the seabed (h).  In the bottom row, changing oceanographic conditions are represented by changes in the current velocity impinging on the curtain, assumed to be constant with depth.  The density contrast across the curtain is assumed to increase linearly with depth below the curtain top at 0.002 (kg/m^3^)/m, up to a maximum of 0.5 kg/m^3^.  To facilitate comparison between curtains with distributed and concentrated buoyancy, the curves for distributed buoyancy are plotted as a function of their per-area buoyancy multiplied by the nominal height of the curtain (250 m).  For the bottom row, we have chosen to plot the variable behavior of the curtain with buoyancy of 10 Mg/m.

In addition to the buoyancy concentrated at the top and evenly distributed along the length of the panels, there is a third alternative of layering the curtain panels with distributed buoyancy concentrated toward the bottom and relatively less buoyancy toward the top.  Panel tensile loads will then be highest at the bottom and progressively lower toward the top. Tapering the panels for greater tensile loading and more buoyancy at the bottom will tend to straighten them while also being more structurally efficient. An approximately quadratic distribution of buoyancy from the top to the base of curtain panels could ideally eliminate curtain curvature in the idealized piecewise linear pycnocline density difference profile of Fig. S1c, which of course is highly approximate and does not allow for the natural large variations in the pycnocline depth and profile.

Oscillation modes associated with curtain curvature are an important fluid-structural interaction issue to be investigated. Large curvature of the panels may encourage large amplitude low frequency vibration modes of concern for stability and fatigue life issues. While hydrodynamic added mass effects would greatly reduce the natural frequency of the lowest longitudinal mode in purely two-dimensional movement, three-dimensional coupled longitudinal and transverse vibrational modes with higher natural frequencies must also be considered.

There is a tradeoff between vertical loads on the foundation and curtain efficacy. Vertical uplift loads on the foundation are more difficult to reinforce against than horizontal loads for all feasible foundation options. Thus, lower-buoyancy curtain options that are closer to horizontal at the base will reduce costs associated with foundation construction. However, the low-buoyancy options are more susceptible to losing height if the ambient seawater conditions change.  An upwelling event in the Amundsen Sea making the thermocline rise will increase the density contrast across the curtain, pushing down a low-buoyancy curtain and increasing spillover of warm water towards the glacier.  A high-buoyancy curtain’s height, by contrast, will be much more stable against fluctuations in the ambient oceanography.  In this context, spending more money on a foundation that can handle larger uplift forces may be viewed as an investment in a more effective curtain.  On the other hand, high-buoyancy curtains may be more vulnerable to damage during iceberg impacts, because increasing the curtain buoyancy will also increase the contact forces between the curtain and the iceberg.  Having more buoyancy near the base and less near the tip may balance between those concerns.

Frontal separation form drag on the curtain may be affected by squeezing of the separation bubble behind the curtain by the overhead outward flow, which itself must overcome internal wave drag resistance from turbulent overturning and mixing. Most studies (e.g. Zhu and Lawrence, 2000) of ocean exchange flows over sills have assumed a relatively gentle and typically Gaussian or trapezoidal sill shape without consideration of flow separation behind the sill, while there will be highly turbulent separation and recirculation behind our curtains leaning steeply toward the glacier side.

1. **Curtain Design Considerations**


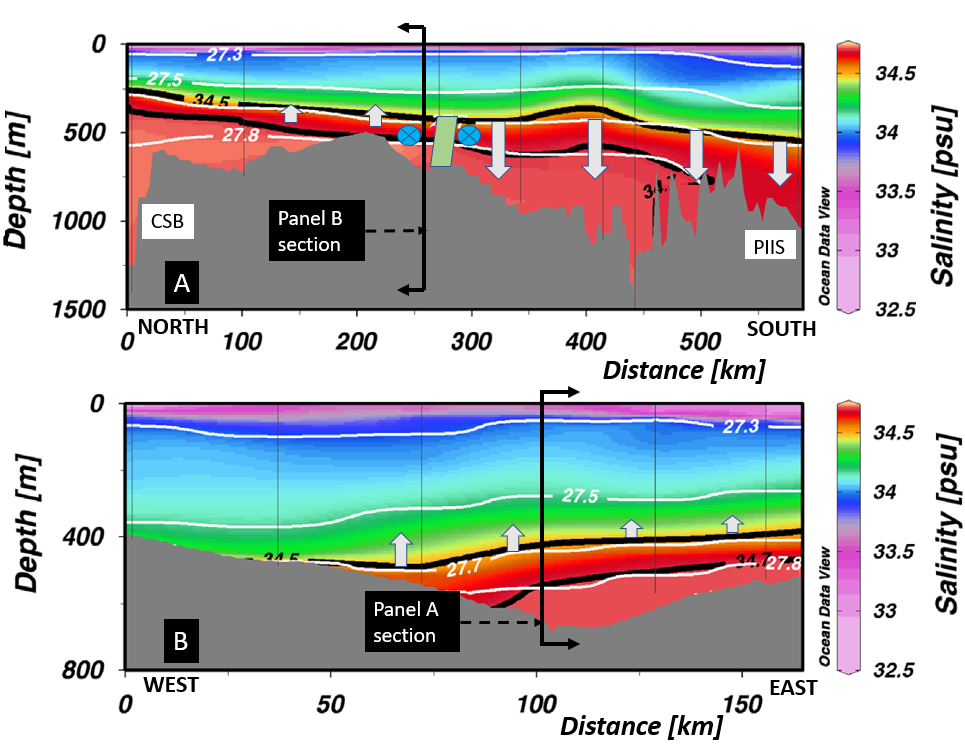


Figure S3. (a) Practical salinity profiles from A) Continental Shelf Break (CSB) to Pine Island Ice Shelf (PIIS), and B) across Pine Island Trough (Fig. 1), shown by colors and thick black contours; surface referenced potential density is indicated by white contours. The vertical black lines mark the location of CTD profiles, from Nakayama et al. (2013). Perpendicular intersection of the two profiles is marked on each panel. Bottom topography is based on Rtopo-1(Timmermann et al., 2010). White arrows show how the pycnocline may respond to a curtain (represented by the light green parallelogram in panel A at approximately the OB route in Fig. 1) and the induced along curtain flow by the blue circles in panel A.

As indicated in Table S1, the tensile loading of the curtains and their foundations will rise very steeply with curtain height above the seabed. Optimal design configurations are likely to be height-dependent and may be quite different for relatively shallow curtains of less than 100 m height, tall curtains of height up to about 250 m, and extremely tall curtains up to about 500 m height.

Foundation design options will be limited by the character of the local seabed ranging between exposed or shallow-buried bedrock, to glacial till or alluvial deposits of clay, sand, gravel, cobbles with occasional boulders. For the contemplated mid-shelf curtain location at 73^o^ S, seabed conditions in the deepest portion of Pine Island Bay trough have been mapped by multibeam swath bathymetry (Jakobsson, 2012; Wise, 2017), showing ice stream bed sediments punctuated by pre-Holocene grounding zone wedges. The largest grounding zone wedge comprises an east-west 50 m high escarpment of moraine deposits which can usefully protect the deepest central portion of a seabed curtain from future ploughing by deep-keeled icebergs. Curtain foundations in this section can readily be provided as rigid frames or caissons secured in position by piles or by cables tensioned to drag embedment anchors.

Seabed conditions on the flanks of the trough have not yet been adequately investigated, but the western flank is underlain by flat-lying Tertiary sedimentary formations, while the eastern flank around Burke Island has older basement formations (Graham et al., 2010) which may be igneous rock with relatively rugged terrain shaped by pre-Holocene glaciation. The shallower flanks are vulnerable to iceberg grounding and ploughing, more severely on the western side northwards of Bear Island where icebergs frequently become grounded. Piles or drag embedment anchors may be feasible in some areas with softer sedimentary cover. In areas with hard rugged rock terrain, the curtain foundation may be custom-contoured to fit the detailed topography, and anchored with rock bolts or drilled and grouted piles.

While curtain sections may be fabricated as relatively short modules delivered by heavy lift or semi-submersible vessels, the remote assembly of much longer sections may facilitate rapid installation in a short working season. These long sections may be assembled in multi-hundred meter or even kilometer lengths, with the curtain panels already attached in a folded or rolled stowage position above the foundation frame. The long curtain sections would then be towed across the ocean in an almost neutrally buoyant condition below streamlined spar buoy floats to minimize ocean surface wave disturbances of the towing operation. Upon reaching the Amundsen Sea, the buoyancy would be carefully adjusted so that the curtain section would float in the middle of the thermocline for delivery to final location without the spar buoy flotation that could be jammed by surface ice.

The risk of catastrophic damage from iceberg ploughing events may be mitigated by designing weak links so that an impacted portion of the curtain may be dislodged from its anchor attachments, pushed aside, and then brought back into location and reattached after the iceberg has passed onward. Spare curtain sections can be held in reserve to close any gaps.

Dynamic vibration absorbers may be configured as buoyancy pendulum or hydropneumatic oscillators tuned to reduce curtain oscillations at critical frequencies. An oscillating water column could be set up in a U-tube between a pair of vertically oriented hard tanks filled with low pressure air to act as gas springs, and the entire assembly having semi-rotary or surging degrees of freedom excited by internal waves in the thermocline. Such devices might be configured as wave energy converters by including small high pressure water pumps (driven by the oscillating water column) as damping elements, with these pumps being driven by curtain oscillations to drain water from hard ballast tanks that provide curtain buoyancy. Such pumps actuated by passive curtain movement might enable a seabed anchored curtain to erect itself by pumping out its own ballast tanks, after installation of curtain panels in an approximately neutrally buoyant condition with the ballast tanks at least partially flooded. A more practicable approach to driving small pumps may harness the water current flowing over the curtain crest, using rotating buoyancy tanks as low efficiency drag turbines.

1. **Installation Methods**

Unloading operations could be conducted in seasonal polynyas that form along the eastern edge of Pine Island Trough (Criscitiello, 2013). Given the ice conditions, precise positioning of a surface lift ship may not be practicable for any extended operations. It may thus be difficult or infeasible to lower negatively buoyant curtain modules vertically to their exact site. Autonomous submersibles with sufficiently powerful thrusters could install guidelines and position neutrally buoyant modules exactly in place. Negatively buoyant modules could be lowered, or positively buoyant modules winched down, along guidelines attached to seabed foundations by remotely operated submersibles. Removable variable buoyancy pontoons may be attached to heavy modules. Buoyancy of the curtain panels or auxiliary pontoons may be lowered by flooding buoyancy chambers, or increased by pumping or using compressed air to blow the water out of those chambers.

Ocean currents will continuously adjust as the curtain is installed. Large transient currents may be controlled by unfolding curtains in stages. As the warmer deep waters are progressively blocked from flowing towards the ice front, reductions in the melt rate will lead to a slowdown in the overturning circulation that drives flow within the ice shelf cavity.

Curtain foundation modules may be delivered and lowered at close to neutral buoyancy if they are intended to be secured in position by deep seabed piles. Seabed curtain modules with heavier gravity foundations may be brought to neutral buoyancy when attached to large self-propelled and dynamic positioning submersibles equipped with adequate variable ballast tank capacity. The construction vessel could also deploy umbilically powered and remotely operated equipment such as seabed bulldozers, subsea piledrivers or rock drills.

In soft alluvial seabed areas, seabed curtain modules could be designed with a sturdy perimeter base skirt which would initially be sunk in the seabed by the module’s submerged self-weight, and then driven deeper by pump-assisted suction.

In areas of rough terrain with hard seabed, drilled and grouted piles or rock anchors may be used, with grouting operations performed with umbilicals from the surface. Unmanned underwater operations may be desirable and have been routine for subsea petroleum wellhead installation and servicing, along with many other deep ocean salvage, seabed coring and oceanographic research applications with no fundamental barrier to scaling up if needed for seabed curtain deployment and maintenance. Remote operated vehicle equipment may be powered by umbilical cables from a surface or submarine support vessel, or by an onboard energy source such as batteries or fuel cells.

**Operational hazards.** The Amundsen Sea presents formidable difficulties in the form of storms, sea ice and drifting ice bergs. Allowances must be made for contingencies and schedule disruptions. Fortunately, the observed drift pattern of icebergs emanating from Pine Island and Thwaites glaciers (Mazur, 2019) has been concentrated in a fairly narrow band on the eastern flank of the central ridge northward from Bear Island, which suggests that installation of a mid-shelf curtain may be feasible despite the prospect of considerably increased iceberg flux. The western end of that alignment is exposed to the iceberg drift track along the ridge flank and may need expensive measures such as seabed trenching and heavily armoured berms for protection against iceberg ploughing. The population density of icebergs may be greatly intensified by accelerating ice shelf fragmentation. The density of icebergs during an episode of full shelf collapse may make any construction work in iceberg congested waters nearly impossible or extremely delayed.

1. **Costs**

At this stage, engineering design concepts for subsea anchored curtains are insufficiently developed and too little is known about seabed conditions for total costs of fabrication and installation to be estimated with great confidence. A preliminary discussion still provides some useful perspectives, with caution as to the uncertainties. Some guidance from somewhat similar projects is provided by very large-scale ocean engineering deployments of offshore wind farms (Katsouris, 2016; Musial, 2003) and pipelines (Kaiser, 2017) in increasingly deep water.

The following categories of costs will apply to construction of an 80 km long seabed anchored curtain at a mid-shelf location to protect both Thwaites and Pine Island Glaciers with an assumed curtain height of 250 m at greatest depth. The construction period is expected to be about 10 years, but on-going maintenance and component replacement would be done each year as detailed below. A full-scale program would almost certainly involve engineering development and learning through smaller projects such as in relatively accessible fjords in Svalbard, Alaska or Greenland which would be funded separately from this project.

1. Project facilities and specialized construction equipment including icebreakers and work submersibles, pile drivers, etc: $10-15b. The capital cost of an advanced icebreaker is about $1b. In our estimate, we assume five such icebreakers, several specialized carrier ships, and associated smaller vehicles and equipment costs.
2. Geotechnical investigations and site seabed preparation: $2-8b. There is a large degree of uncertainty in this component of the cost, some foundation designs and seabed conditions will need little if any site preparation, while others on rough bedrock will require much more. The upper end of our uncertainty range corresponds to $0.1b per km of curtain length.
3. Curtain foundations including pile or drag embedment anchors based on designs for offshore windfarms (Katsouris, 2016; Musial, 2003): $6-10b. Drag embedment anchors would cost substantially less than piles, but only work with certain seabed conditions.
4. Curtain panel fabrication: $10-15b. We are assuming the use of high modulus polyethylene (HMPE) for fabric and tensile components because of its superior performance attributes, though its present cost in low volume production 8 times more expensive (Yeroshina, 2017) than more common polyester material which may be adequately suitable. HMPE and related grades of ultra-high molecular weight polyethylene are used for marine mooring ropes, marine fenders, bridge bearings, premium grade nets, racing yacht sails and penetration-resistant personal armour. These materials are nearly neutrally buoyant, entirely compatible with long term seawater immersion, and biologically inert. We are assuming a thick cross-ply laminated curtain structure for highest durability. Materials cost will drop with the high manufacturing volumes needed to deliver the approximately 16 km^2^ of curtain area including panel overlaps, or if less expensive materials are used.
5. Installation and curtain deployment: $1.25-2.5b. Assuming 100-day working seasons each year and equipment running costs estimated from shipping charters comes to about $2m/day or $2b for a decade long installation. Alternative analogues for installation come from operational and logistical costs for major Antarctic operators such as British Antarctic Survey (BAS) and Alfred-Wegener Institute (AWI). BAS annual budget is about $50m/yr of which less than half is for ship and aircraft operations, so we assume $25m/yr (Natural Environment Research Council Annual Report and Accounts 2017-18 – the most recent available). BAS run 2 ice strengthened research ships. 5 aircraft, 5 polar bases, I UK base. Scaling up to 5 icebreakers and 10 support ships means by a factor of 5-10, or $125-250m/yr. A 10-year installation is then $1.25-2.5b, in reasonable agreement with the estimate from chartering.
   We note that iceberg disruptions may require operations to concentrate at one end of the 80 km curtain while the other end is inaccessible, and that seasonal shifts of project vessels and crews between Antarctic and Arctic waters would improve the annual operational capacity factor.
6. Management, engineering and rear base support: $1-1.6b. If we take BAS and AWI as examples and scale appropriately to around $100m/yr. Probably less relevant are the typically 3.2% of the total cost is reserved in large offshore wind farms (<https://guidetoanoffshorewindfarm.com/wind-farm-costs>) which is about 50% larger.
7. Contingencies: schedule disruptions by ice conditions may to some extent be absorbed in a 10-year schedule for installation with good working seasons being offset by poor ones. But unknown unknowns might be assumed to be 1/4-1/2 of total project costs.

**Total curtain construction costs** on the above assumptions would be $60±10b (expected plus minus uncertainty) or as full range $38b to $78b, averaging **$4b to $8b per year** over the assumed 10-year construction period.

1. Annual inspection and maintenance, plus forward-located inventory of curtain replacement spares. This comprises several elements: we have A=100 day season construction fleet running costs. We assume maintenance would require only 20% of the build fleet effort, so cost A/5; curtain panel fabrication costs, C, spread over a 30 year lifetime, result in an ongoing cost of C/30 per year; doubling this to allow for iceberg damage produces C/15 per year; foundation costs, F, with 100 year lifetime, and again doubled for damage, results in F/50 per year; specialized construction equipment and ships, S, with lifetime of 10 years, would be S/10 per year, but this would be an overestimate since we assume that repairs may be done with 20% of the build fleet and management costs, yielding a final estimate of S/50 per year. Thus, we estimate annual maintenance costs based on our above estimates of initial costs as, A/5+C/15+F/50+S/50. Taking the above figures, adding 25% to the high end costs for more contingencies, the ongoing annual maintenance and refurbishment cost would be about **$1-2 b/yr**.

The above estimates are based on plausibly feasible conceptual design approaches and incomplete site information, which must be elaborated much more rigorously during feasibility studies. We expect that there will be opportunities for cost reduction from design improvements and efficiencies of high manufacturing volumes, but also recognize the downside risks of ice-related accidents and major schedule disruptions resulting in cost escalation.

1. **Research Priorities**

Comprehensive feasibility investigations will include the following components. The first a) through i) refer to understanding more about the subsea curtains, the remaining ones are likely to be done as part of wider research into the Amundsen Sea region.:

1. Modelling of coupled oceanographic and ice dynamic response to deployment of curtains at each envisaged site for a range of nominal installed curtain heights and a range of effective large-scale curtain permeabilities (or curtain leakiness).
2. Modelling and analysis of fluid-structural interactions, oscillation modes and potential instabilities for alternative curtain configurations and tensioning modes.
3. Program cost and schedule estimates.
4. Analysis of risk factors.
5. Investigation of environmental and ecological impacts, including local and far-field effects, both in Antarctica and in fabrication facilitiesTank tests of curtain dynamics in stratified exchange flow with rotational effects.
6. Tank tests of simulated iceberg-curtain collisions
7. Curtain design definition, including materials selection, structural analysis and testing.
8. Foundation design definition, including gravity foundations, anchors or piles for expected seabed conditions, and scour protection where needed.
9. Investigation of installation and maintenance procedures and necessary equipment.
10. Intensification and expansion of glacio-oceanographic data collection like that collected in the present International Thwaites Glacier Collaboration, including current patterns, internal waves, ice cover and iceberg drift trajectories.
11. Ongoing projections of ice shelf retreat and rifting instabilities in the subject area, with attention to risk factors of accelerating ice shelf collapse and discharge of deeper keeled icebergs.
12. Geotechnical investigations, including seabed contours, composition and coring along prospective curtain alignments.

**Supplemental References**

Dong, Z., Mu, Q., Luo, W., Qinan, G Lu, P.and Wang, H.: An analysis of drag force and moment for upright porous wind fences, J. Geophys. Res. 113, 04103 (2008) doi: 10.1029/2007JD009138

Graham, A., Larter, R., Gohl, K., Dowdeswell, J., Hillenbrand, C., Smith, J., Evans, J., Kuhn, G. and Deen, T.: Flow and retreat of the late Quaternary Pine Island-Thwaites palaeo-ice stream, West Antarctica, Journal of Geophysical Research 115, F03025 (2010)

Jakobsson, M., Anderson, J., Nitsche, F., Gyllencreutz, R., Kirshner, A., Kirchner, N., O’Regan, M., Mohammed, R., Eriksson, B.: Ice sheet retreat dynamics inferred from glacial morphology of the central Pine Island Bay trough, West Antarctica, Quaternary Science Reviews 38, 1-10 (2012)

Kaiser, M.: Offshore pipeline construction cost in the U.S. Gulf of Mexico, Marine Policy 82, 147-166 (2017)

Katsouris, G. and Marina, A.: Cost modelling of floating wind farms, Energy research Centre of the Netherlands, report ECN-E-15-078 (2016)

Mazur, A., Wahlin, A. and Kalen, O.: The life cycle of small- to medium-sized icebergs in the Amundsen Sea Embayment, Polar Research 38, 3313-3329 (2019)

Musial, W., Butterfield, S. and Boone, A.: Feasibility of floating platform systems for wind turbines, NREL/CP-500-34874, National Renewable Energy Laboratory, preprint for 23^rd^ ASME Wind Energy Symposium (2003)

Nakayama, Y., Schroder, M. and Hellmer, H.H.: From circumpolar deep water to the glacial meltwater plume on the eastern Amundsen Shelf. Deep Sea Research Part I 77:50–62 (2013)

Pratt, L.: Hydraulic control of sill flow with bottom friction, Journal of Physical Oceanography 16, 1970-1980 (1986)

Timmermann, R., Brocq, A.L., Deen, T., Domack, E., Dutrieux, P., Galton-Fenzi, B., Hellmer, H., Humbert, A., Jansen, D., Jenkins, A., et al.: A consistent data set of Antarctic ice sheet topography, cavity geometry, and global bathymetry. Earth Syst. Sci. Data 2, 261–273 (2010)

Vlasblom, M., Boesten, J., Leite, S., Davies, P.: Creep and stiffness of HMPE fiber for permanent deepwater offshore mooring, IEEE Oceans Conference, Yeosu (2012)

Wise, M., Dowdeswell, J., Jakobsson, M. and Larter, R.: Evidence of marine ice-cliff instability in Pine Island Bay from iceberg-keel plough marks, Nature 550 (7677) 506-510 (2017)

Yeroshina, S.: Cost-effectiveness of sailcloth selection for different classes, types and species of sailing boats, Eureka: Physics and engineering 5, 22-29 (2017)

Zhu, D. and Lawrence, G.: Hydraulics of exchange flows, Hydraulic Engineering 126 (12) 921-926 (2000)
